# An unusual case of pedunculated subserosal leiomyosarcoma of the uterus mimicking ovarian carcinoma

**DOI:** 10.1186/s13048-016-0212-4

**Published:** 2016-02-09

**Authors:** Dong Soo Suh, Yoon Hwa Kim, Ka Yeong Yun, Nam Kyung Lee, Kyung Un Choi, Ki Hyung Kim, Man Soo Yoon

**Affiliations:** 1 grid.262229.f0000000107198572Department of Obstetrics and Gynecology, Pusan National University School of Medicine, 179, Gudeok-Ro, Seo-Gu, Busan, 602-739 Korea; 2 grid.412588.20000000086117824Biomedical Research Institute and Pusan Cancer Center, Pusan National University Hospital, 179, Gudeok-Ro, Seo-Gu, Busan, 602-739 Korea; 3 grid.262229.f0000000107198572Department of Radiology, Pusan National University School of Medicine, and Biomedical Research Institute, Busan, 602-739 Korea; 4 grid.262229.f0000000107198572Department of Pathology, Pusan National University School of Medicine, and Biomedical Research Institute, Busan, 602-739 Korea

**Keywords:** Leiomyosarcoma, Postmenopausal, Pedunculated

## Abstract

**Background:**

Leiomyosarcoma of the uterus is an extremely rare but highly aggressive tumor that accounts for only 1–2 % of uterine malignancies, and is usually associated with a dismal outcome.

**Case presentation:**

The authors present an unusual case of pedunculated subserosal leiomyosarcoma of the uterus mimicking ovarian carcinoma. A 57-year-old postmenopausal woman presented with progressive low abdominal pain and urinary frequency. Pelvic magnetic resonance imaging revealed a large adnexal mass with carcinomatosis peritonei. Laboratory examination revealed an elevated serum CA-125 level (398.4 U/ml). The patient underwent exploratory laparotomy under suspicion of ovarian malignancy. Frozen section indicated malignancy originating from the uterus, and thus, total abdominal hysterectomy, bilateral salpingo-oophorectomy, omentectomy, pelvic and para-aortic lymph node dissection, and mass excision were performed. Subsequent histopathologic examination resulted in a final diagnosis of leiomyosarcoma of the uterus. The patient’s postoperative course was uneventful, and gemcitabine and docetaxel adjuvant chemotherapy was administered.

**Conclusion:**

The authors report an unusual case of pedunculated subserosal leiomyosarcoma of the uterus mimicking ovarian carcinoma.

## Background

Uterine leiomyosarcoma (LMS) is a rare uterine malignancy that accounts for approximately 1–2 % of uterine malignancies and 25–36 % of uterine sarcomas. It occurs mainly after menopause (mean age at diagnosis is 60 years [1]) and usually presents as abnormal uterine bleeding [2]. LMS is highly aggressive tumor with a poor prognosis; a review of the literature showed that recurrence rates range from 45 to 73 % and 5-year overall survival rates between 30 and 42 % [3]. Here, we report an unusual case of pedunculated subserosal leiomyosarcoma of the uterus mimicking ovarian carcinoma, and describe its imaging findings in a postmenopausal woman.

## Case presentation

**Fig. 1:**
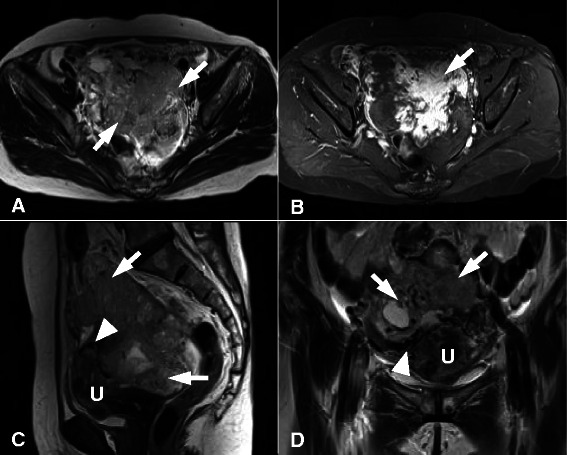
Pelvic magnetic resonance image of the pedunculated subserosal leiomyosarcoma of the uterus. **a** Axial T2-weighted image showing a large pelvic mass (arrows) with heterogeneous signal intensity. **b** Contrast-enhanced fat-suppressed T1-weighted image showing marked heterogeneous enhancement (arrow) of the mass. **c**, **d** On sagittal (**c**) and coronal (**d**) T2-weighted images, the mass (arrows) abutted the uterus (U). Note the bridging vessels (arrowheads) between the uterus and the juxta-uterine mass

A 57-year-old postmenopausal woman presented with progressive low abdominal pain and urinary frequency of 4 months duration. The patient had no significant gynecological, medical, surgical, or family history, and had no associated bowel complaints. Physical examination revealed an enlarged, relatively nodular pelvic mass, and pelvic ultrasonography and magnetic resonance imaging (MRI) depicted a large, complex, adnexal mass, suggestive of ovarian carcinoma. Axial T2-weighted imaging showed a solid, cystic mass with heterogeneous signal intensity in the pelvic cavity. Contrast-enhanced fat-suppressed T1-weighted imaging showed heterogeneously marked enhancement of the mass. On sagittal and coronal T2-weighted images, the mass abutted the uterus (Fig. 1). Ascites and omental smudging were suspected to be due to carcinomatosis peritonei. There was no significant lymphadenopathy. Laboratory examination revealed a raised serum tumor marker (CA-125, 398.4 U/ml) level. Urinalysis results were normal. At this juncture, based on imaging findings and the elevated serum tumor marker level, an ovarian malignancy was suspected.

**Fig. 2:**
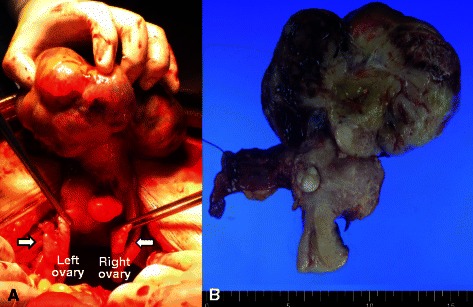
**a** Gross appearance of pedunculated subserosal leiomyosarcoma of the uterus. The mass was bilobed rather than bilateral. Both ovaries were normal in appearance. **b** Cut section of the uterus and tumor

**Fig. 3:**
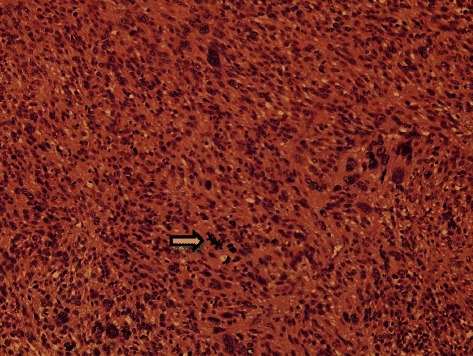
The tumor consisted of highly atypical spindle cells, which exhibited atypical mitosis and fascicular arrangements (arrow) (H&E, ×200)

**Fig. 4:**
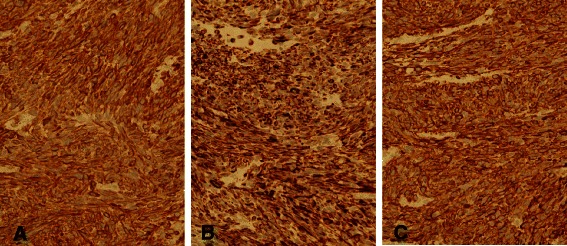
Immunohistochemistry revealed diffuse strong positivity for SMA (**a**), desmin (**b**), and h-caldesmon (**c**) (×400). SMA: smooth muscle actin

The patient underwent exploratory laparotomy under suspicion of ovarian malignancy. A huge pedunculated subserosal mass arising from the uterine fundus in the cul-de-sac was confirmed that was bilobal rather than bilateral, and. The mass was adherent to omentum and rectosigmoid. Both ovaries were normal in size and contour (Fig. 2), and there was moderate amount of ascites. A portion of the mass was sent for frozen section, which revealed malignancy originating from the uterus. Total abdominal hysterectomy, bilateral salpingo-oophorectomy, omentectomy, pelvic and para-aortic lymph node dissection, and excision of a metastatic mesenteric mass of diameter 3.0 cm were performed. The cut tumor surface was soft, necrotic, and hemorrhagic with myxoid changes (Fig. 2), and the omentum showed multiple brown-white solid nodules. Microscopically, the tumor was composed of highly atypical spindle cells with brisk mitotic activity (≥28/10 high-power fields) and necrosis (Fig. 3). Omentum and mesentery around the rectosigmoid showed metastatic disease, confirming stage III disease. Pelvic and para-aortic lymph nodes were free from tumor cells, and peritoneal washings were negative for malignant cells. Immunohistochemical staining showed tumor cells were positive for smooth muscle actin (SMA), desmin, and h-caldesmon, and negative for CD10, ER, PR, inhibin, and PanCK (Fig. 4). Her postoperative course was uneventful. Gemcitabine and docetaxel adjuvant chemotherapy was administered and serum CA-125 decreased to normal at 1 month after surgery. No recurrence occurred over 17 months of postoperative follow-up.

## Discussion

Leiomyosarcoma (LMS) of the uterus is an extremely rare but highly aggressive tumor and is associated with poor prognoses, even for early-stage disease. LMS is usually a large, yellow or tan solitary myometrial mass with some hemorrhagic and necrotic areas. Necrosis, moderate-to-severe cytologic atypia, and > 15 mitotic figures per 10 high power fields (HPF) are considered typical histopathologic criteria. It may be near impossible to differentiate benign and malignant uterine tumors; however, pathological features, such as, larger tumor cells, fewer stromal fibers, increased mitotic activity, and nuclear pleomorphism may aid differentiation. Nevertheless, the presence of mitoses is a hallmark of malignancy.

The signs and symptoms of LMS are similar to those of benign leiomyoma. Presenting symptoms are nonspecific and include abnormal vaginal bleeding (also a common presentation of uterine sarcoma), abdominal pain, vaginal discharge, urinary frequency, constipation, and abdominal distention. However, in our case, the patient had no complaint of vaginal bleeding possibly because of an unusual subserosal location.

Serum CA125 levels are occasionally elevated in LMS. It has been reported preoperative serum CA125 levels are significantly higher in LMS than in uterine leiomyoma [4]. Although the cause of this elevation has not been determined, Duk et al. suggested that CA125 elevation in LMS is probably caused by irritation of epithelial surfaces by tumor cells rather than by tumor tissues *per se* [5]. In our case, serum CA-125 was considerably elevated (398.4 U/ml and given the presence of an adnexal mass, a diagnosis of primary ovarian carcinoma was made.

MRI is very useful for determining anatomic features, such as, the locations, sizes, and numbers of pelvic masses. In addition, MRI may provide diagnostic information that supports a preoperative presumption of leiomyosarcoma; however, it is not entirely accurate. LMS usually manifests as a large infiltrating myometrial mass with high signal intensity on T2W images and low ADC values. Final diagnosis of uterine leiomyosarcoma is made by histopathological findings. In our case, an incorrect initial diagnosis of ovarian malignancy was made because ovarian cancer is more common in postmenopausal women and MRI showed a large, adnexal mass, ascites, omental smudging, and misty mesentery.

Pedunculated masses may have obscure origins and be misinterpreted as ovarian masses. The detection of a vascular pedicle can provide information on the origin of a mass. When normal ovaries are visualized separately, a mass of ovarian origin can be excluded. In our case, we retrospectively analyzed MR images, and noted bridging vessels between the uterus and a juxta-uterine mass, suggesting a pedunculated subserosal uterine mass. However, normal ovaries were not visualized because ovaries were atrophied and obscured by the mass. Several case reports have been issued on uterine masses considered preoperatively to be ovarian tumors, due to similar locations and imaging findings. For example, a large pedunculated leiomyoma with cystic degeneration mimicking ovarian malignancy [6], and an unusual huge, multiseptated, pedunculated pyomyoma occupying the entire abdomen that was initially misdiagnosed as ovarian cancer based on imaging findings have been reported [7]. Furthermore, in postmenopausal women, uterine leiomyoma with hemorrhagic cystic degeneration mimicking ovarian malignancy [8] and leiomyosarcoma of the rectum mimicking primary ovarian carcinoma with normal serum marker levels and no rectal bleeding [9] have been reported. However, few reports have been issued on pedunculated subserosal leiomyosarcoma of the uterus simulating ovarian carcinoma in terms of anatomic location, imaging findings, or serum tumor marker level.

Immunohistochemistry may be helpful for differentiating leiomyosarcoma from other tumors. In our case, immunohistochemistry revealed diffuse strong positivity for smooth muscle actin (SMA), desmin and h-caldesmon, and SMA and desmin are usually expressed in LMS, whereas h-caldesmon is highly specific for LMS [10].

Total hysterectomy and bilateral salpingo-oophorectomy are recommended for LMS grossly confined to the uterus, but dissection of pelvic and para-aortal lymph nodes is not routinely recommended. Lymphatic spread is not common and lymph node involvement is only encountered in <3 % [11]. However, the presence of metastatic disease indicates the need for complete surgical cytoreduction. Furthermore, lymphadenectomy should be performed in patients with suspicious metastatic nodes or extrauterine disease. Nonetheless, despite aggressive cytoreductive surgery, intraperitoneal recurrence or distant metastasis occurs in >50 % of cases [12].

LMS is characterized by early dissemination and metastases to multiple organs and primarily presents with hematogenous spread. Interestingly, in the described case, metastases occurred to omentum and mesentery around the rectosigmoid via direct peritoneal spread from the pedunculated subserosal leiomyosarcoma. Prognosis primarily depends on the extent of disease at diagnosis and mitotic index. In addition, several authors have suggested that tumor size may be an important prognostic factor [2, 13]. Garcia et al. [3] reported rates of recurrence or progressive disease of 76 and 85 % for stage I and stages II–IV, respectively, and overall survivals for stage I of 64 and 38 % at 3 and 5 years (overall survival was 30 % for stages II–IV). Although the role of adjuvant chemotherapy is unclear, patients with advanced-stage disease are recommended for gemcitabine and docetaxel adjuvant chemotherapy, for which the response rate is around 36 % [14, 15].

## Conclusion

In summary, we present an unusual case of pedunculated subserosal leiomyosarcoma of the uterus that mimicked ovarian malignancy, based on anatomic location, imaging findings, and serum tumor marker level, in a postmenopausal woman. This case cautions pedunculated subserosal leiomyosarcoma of the uterus with a highly elevated serum CA-125 marker may mimic ovarian carcinoma and should be considered in the differential diagnosis of an adnexal mass in postmenopausal women.

## Consent

Written informed consent was obtained from the patient for publication of this case report and any accompanying images. A copy of the written consent is available for review by the Editor of this journal.

## Abbreviations

CA-125: carbohydrate antigen 125
ER: estrogen receptor
LMS: uterine leiomyosarcoma
MRI: magnetic resonance imaging
PR: progesterone receptor
SMA: smooth muscle actin
